# SG-APSIC1182: Sequential time workforce management

**DOI:** 10.1017/ash.2023.100

**Published:** 2023-03-16

**Authors:** Nanthipha Sirijindadirat

**Affiliations:** President, Central Sterilizing Services Association, Bankok, Thailand

## Abstract

**Objectives:** Since 2018, the workload in the central sterilization services department (CSSD) has intensified as surgeries have increased. The extended operative time among complicated surgical cases has also led to shortages of resterilized surgical instruments. One factor influencing these shortages was inadequate CSSD staff during high workload periods. We developed a strategy to improve the availability of resterilized instruments. We sought to reduce wasted time and improve effectiveness of surgical instrument preparation by adopting a shift-work arrangement. We additionally sought to minimize unorganized instrumentation and surgical equipment loss. **Methods:** Team members investigated workload disproportion shift by shift. We devised a practical arrangement of staff for each work shift by dividing manpower in ratios based on workload. **Results:** The period from 10:00 a.m. to 7:00 p.m. was the period of most intense workload in the CSSD. However, 3 staff worked the morning shift and 2 staff worked the evening shift (4:00 p.m.– 2.00 a.m.). We reassigned 1 person to work from 8.00 a.m. to 4.00 p.m. and 2 persons for an extra shift from 10:30 a.m. to 6:30 p.m. After the manpower readjustment, surgical equipment damage and loss decreased from 57 to 26 losses per year from 2018 to 2021. In addition, work productivity increased from 85% to 115%. Worker satisfaction improved >70%. **Conclusions:** Internal inconsistency concerning instrumental preparation and improper instrument arrangement can affect surgery time. By addressing workload and shift distribution of labor, productivity notably improved, with higher satisfaction and a dramatic decrease in surgical equipment loss.

